# Microfluidics: A Groundbreaking Technology for PET Tracer Production?

**DOI:** 10.3390/molecules18077930

**Published:** 2013-07-05

**Authors:** Christian Rensch, Alexander Jackson, Simon Lindner, Ruben Salvamoser, Victor Samper, Stefan Riese, Peter Bartenstein, Carmen Wängler, Björn Wängler

**Affiliations:** 1 GE Global Research, Freisinger Landstrasse 50, Garching bei Munich 85748, Germany; E-Mails: salvamoser@ge.com (R.S.); victor.samper@research.ge.com (V.S.); 2 GE Healthcare, Life Sciences, The Grove Centre, White Lion Rd., Amersham HP7 9LL, UK; E-Mails: alex.jackson@ge.com (A.J.); stefan.riese@ge.com (S.R.); 3 University Hospital Munich, Department of Nuclear Medicine, Ludwig Maximilians-University, Munich 81377, Germany; E-Mails: Simon.Lindner@med.uni-muenchen.de (S.L.); peter.bartenstein@med.uni-muenchen.de (P.B.); Carmen.Waengler@med.lmu.de (C.W.); 4 Biomedical Chemistry, Department of Clinical Radiology and Nuclear Medicine, Medical Faculty Mannheim of Heidelberg University, Mannheim 68167, Germany; 5 Molecular Imaging and Radiochemistry, Department of Clinical Radiology and Nuclear Medicine, Medical Faculty Mannheim of Heidelberg University, Mannheim 68167, Germany

**Keywords:** microfluidics, PET, molecular imaging, PET probes, PET tracers, PET biomarkers, radiolabelling, synthetic chemistry, lab on a chip, radiochemistry, probe discovery

## Abstract

Application of microfluidics to Positron Emission Tomography (PET) tracer synthesis has attracted increasing interest within the last decade. The technical advantages of microfluidics, in particular the high surface to volume ratio and resulting fast thermal heating and cooling rates of reagents can lead to reduced reaction times, increased synthesis yields and reduced by-products. In addition automated reaction optimization, reduced consumption of expensive reagents and a path towards a reduced system footprint have been successfully demonstrated. The processing of radioactivity levels required for routine production, use of microfluidic-produced PET tracer doses in preclinical and clinical imaging as well as feasibility studies on autoradiolytic decomposition have all given promising results. However, the number of microfluidic synthesizers utilized for commercial routine production of PET tracers is very limited. This study reviews the state of the art in microfluidic PET tracer synthesis, highlighting critical design aspects, strengths, weaknesses and presenting several characteristics of the diverse PET market space which are thought to have a significant impact on research, development and engineering of microfluidic devices in this field. Furthermore, the topics of batch- and single-dose production, cyclotron to quality control integration as well as centralized versus de-centralized market distribution models are addressed.

## 1. Introduction

Microfluidic Positron Emission Tomography (PET) [1] tracer synthesizer development has been pursued for several years [2,3,4,5,6,7,8]. Numerous microfluidic devices have been described, including commercially available capillary-based microfluidic synthesis platforms [9,10,11], as well as lab-on-chip devices [12]. Both approaches have demonstrated significant improvements to PET tracer synthesis such as reduced reaction times, lowered consumption of expensive reagents and processing of radioactivity levels sufficient for practical use [13]. Results published by our group suggest that adverse side-effects resulting from high radioactivity concentrations due to reagent volume down-scaling towards microfluidic dimensions can be circumvented by appropriate micro-reactor design [14]. From the literature it can be concluded that the application of microfluidics to the synthesis of PET tracers as well as its scientific and economic value have been successfully demonstrated. However, future commercial microfluidic PET tracer synthesis systems must deliver the advantages of microfluidics while competing against automated chemistry modules already established in the field. These conventional systems have overcome practical challenges such as regulatory compliance with operation under Good Manufacturing Practice (GMP) guidelines, high operational efficiency and reliability, ease of use and low cost of consumables. In this context, the overall system design and the choice of materials utilized for cassette, chip and reagent kit manufacturing are of fundamental importance. This review takes an engineering perspective on the field of microfluidic PET tracer synthesis and sheds light on achievements and remaining challenges, as well as different perspectives towards making microfluidics for PET radiochemistry a success story worldwide.

## 2. PET Tracer Supply Chain and Synthesis Workflow

### 2.1. PET Tracer Production Workflow

**Figure 1:**
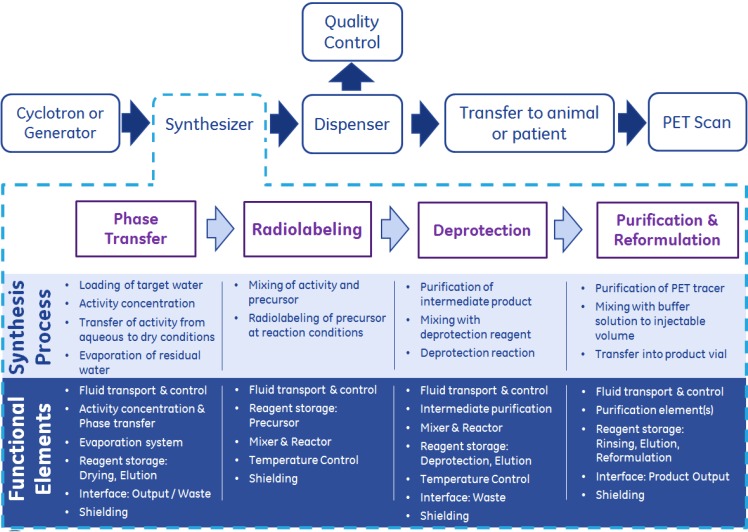
PET tracer production and synthesis workflow and resulting functional elements to be integrated into a microfluidic synthesis system. Synthesizer process description based on [^18^F]FDG synthesis, several functional elements may not be required for the synthesis of other PET tracers.

An overview on the PET tracer production process is schematically illustrated in the upper half of Figure 1.

The workflow focuses on the fluoride-18 based radiochemistry. In the first step (1) “Cyclotron or Generator”, the short-lived (t = 109.8 min) radioisotope fluoride-18 is produced via proton bombardment of enriched [^18^O]H_2_O water by the ^18^O(p, n)^18^F nuclear reaction. The cyclotron bombardment targets usually facilitate a [^18^O]H_2_O target volume of about 1 to 3 mL. A few medical radionuclides can be produced by an activity generator which utilizes a long-lived parent isotope that decays to the medical isotope of interest, omitting the need for a cyclotron. For the example of Ga-68, the parent isotope is Ge-68, with a half-life of 271 days. Upon completion of step (1), this volume is partially or entirely transferred to the PET tracer synthesizer. This transfer can be executed directly via tubing connecting the cyclotron and the synthesizer or by means of an intermediate transport vessel which can be shipped from the cyclotron facility to the location of the synthesizer. In step (2) “Synthesizer”, the radioisotope is directly or via intermediate steps reacted with the precursor. The output of this process is the PET tracer, usually formulated with a saline buffer solution to a total volume of 6 to 14 mL. Depending on the amount of radioactive isotopes utilized during the synthesis, the output dose can serve from one (single dose) to multiple (batch mode) patients. In step (3) “Dispenser”, the batch mode produced PET tracer solution is split into aliquots serving multiple patients. However, additional aliquots have to be provided for the quality control process. In step (4) “Quality Control”, the PET tracer is validated to be suitable for injection to a human patient. Usually, Pharmacopoeia monographs are used to determine the required specifications, including tests on pH, chemical purity, residual solvent, radionuclide purity, radiochemical purity, radioactive concentration, specific activity, sterility, bacterial endotoxins and filter membrane integrity [15]. These quality control tests are often carried out in a dedicated quality control (QC) laboratory. Step (5) “Transfer to animal or patient” describes the logistics of delivering each PET tracer dose to the respective animal subject (research and clinical studies) or to the human patient (clinical practice) in order to execute process step (6), the “PET scan”. In the case of in-house, also referred as to “decentralized” PET tracer production (within a single facility or site), this transfer can be fast and effective. For centralized PET tracer production at a radiopharmaceutical facility and subsequent shipping to hospitals within e.g. two hours driving distance, technical, economic and practical challenges arise concerning activity loss and decrease in specific activity due to transfer time. The geographical distance between the PET tracer production facility and the customer hospitals in low *versus* high density populated areas, as well as patient scheduling at point of use, all need to be considered. For the radioisotope carbon-11 with a half-life of only 20.3 min, the time associated with PET tracer distribution from a central production site to surrounding hospitals renders a centralized production approach infeasible [16]. In the future, the variety of PET tracers targeting specific disease patterns will grow since academic and commercial entities worldwide are developing new compounds today. This will apply pressure to existing PET tracer production sites to expand their capabilities.

### 2.2. PET Tracer Synthesis Process

The PET tracer synthesis process is executed during step (2) “Synthesizer” of the PET imaging workflow (Figure 1). It is displayed exemplarily following the routine for fluoride-18 labeled PET tracers via direct one-step labeling using a nucleophilic substitution such as [^18^F]fluoro-2-deoxy-d-glucose ([^18^F]FDG) [17] or [^18^F]fluorothymidine ([^18^F]FLT) [18]. The process can be separated into four steps: (1) concentration and phase transfer of fluoride-18 activity from the cyclotron target water into dry conditions, (2) radiolabeling of the precursor, (3) deprotection of the radiolabeled precursor via e.g. hydrolysis and subsequent (4) purification & reformulation into a single or multi patient batch dose. Each process step results in elements required for physical implementation into a synthesizer device, herewith referred as to “functional elements” (Figure 1).

### 2.3. Continuous-Flow & Batch Microfluidics

Microfluidic systems can be separated into continuous-flow and stop-flow (batch mode) architectures [19,20,21]. This definition is driven by a perspective towards chemical processing. Continuous-flow architectures perform all processing steps such as mixing, heating and cooling along a pre-defined fluid path whereby all reagents progress continuously through the system. Parameters such as exposure time to reaction temperatures are defined by the reagent flow rate with respect to the fluid path geometry. Design advantages are the straightforward architecture and the large flexibility in processing small or large product quantities on the same device. Not surprisingly, many early studies on microfluidic labeling of PET tracers were carried out on fluoride-18 chemistries following the continuous flow approach [22,23,24,25]. Comparable examples can be found for the microfluidic synthesis of iodine-124, copper-64, nitrogen-13, technetium-99m and carbon-11 labeled compounds [26,27,28,29,30,31,32]. Disadvantages of continuous-flow microfluidics are the strong dependency on precise reagent flow control, long flow paths with a high accumulated contact area between reagents and the fluid path material resulting in high system pressures and increased reagent to fluid path material interactions (residual activity), the risk of channel clogging and limited functionality integration of multiple reaction steps. Pressures in continuous-flow systems depend on reagent viscosities, applied flow rates, dimensions and system architecture. Although high pressures are advantageous for certain reactions, pressures in excess of 100 bar can occur and this places additional demands on components to avoid leakages and syringe pump stalls. A recent review on microfluidics in radiochemistry proposes the term “micro-channel systems” (MCSs) which unites capillary-based and chip-based continuous flow architectures [33].

Batch-mode or stop-flow microfluidics employs discontinuous flow rates of reagents, usually due to mixing or reaction of starting compounds or intermediates within one or multiple functional elements. Another description proposed by Pascali *et al*. describes this category as “micro-vessel systems” (MVSs), implying a straightforward downscaling approach from conventional, vessel-based synthesizers [33]. In this particular architecture, e.g., temperature exposure intervals are usually driven by residence time at stopped flow. There are several examples of batch-mode PET tracer synthesis devices [34,35]. A high level of functionality integration is often seen as an important advantage of batch-mode microfluidics over continuous-flow architectures [21]. Consequently, the hardware structure of batch-mode microfluidic systems is rather complex with challenges on functional element integration, manufacturing, hardware assembly and system control.

### 2.4. Capillary-Based, Hybrid and Integrated Lab-on-Chip Systems

Another angle from which to look at microfluidic architectures is from the perspective of hardware engineering, categorizing state of the art microfluidic systems into: (1) capillary-based microfluidic systems, (2) hybrid assemblies and (3) chip-based integrated devices. Commercial examples for capillary-based microfluidic PET tracer synthesizers are the Advion Biosciences NanoTek (Advion Biosciences, Inc., Ithaca, NY, USA) and the Scintomics “μ-ICR” (SCINTOMICS GmbH, Fuerstenfeldbruck, Germany). Both solutions and modifications thereof are often associated to continuous-flow operation and show a comparably simple hardware structure utilizing conventional syringe pumps and rotary motor valves as well as established fluid connectors and capillaries. Presenting a low development risk, these systems were consequently the first to enter the commercial space. Capillary-based synthesizers have demonstrated numerous microfluidic benefits to PET tracer syntheses such as low reaction volume processing, reduced reaction times, high radiolabeling yields and efficient screening of multiple reaction conditions [36,37,38,39,40,41,42,43,44,45,46,47,48,49,50,51,52]. A summary on chemistry results accomplished utilizing the Advion Biosciences, Inc. NanoTek capillary-based synthesis system has recently been published [33].

The second category, hybrid assemblies, describes a transient state of technology development bridging conventional hardware concepts and highly integrated microfluidic “lab-on-chip” systems. There are several example systems which combine conventional aspects such as macroscopic valves, syringe pumps and turbulent mixing of reagents with new monolithic microfluidic chip devices or chip-like assemblies including the respective manufacturing technology [34,53,54,55,56,57,58,59]. The third category, chip-based integrated devices, can be described by a high level of functionality integration into a microfluidic core component. Examples are a highly integrated PDMS-based [^18^F]FDG processing system and a “electrowetting-on-dielectric” (EWOD) chip applied to PET tracer synthesis [60,61,62]. The high level of innovation and the associated development risk of this third hardware category is driven by the need for new technologies for functional elements and materials, as well as manufacturing processes, whilst a path towards regulatory compliance has not been defined yet.

### 2.5. Reusable and Disposable Fluid Paths

PET tracer synthesizers use either a reusable or a disposable fluid path (reaction vessels, tubes, valves, *etc.*). Utilization of a disposable fluid path (“cassette”) that is replaced after each synthesis run avoids the need for fluid path cleaning and validation of the cleaning process itself. The cleaning procedure may depend on the specific PET tracer produced in the synthesis run of concern, as well as previous synthesis runs. Therefore, replaceable cassette based synthesizers have become widely used for routine production of clinically used tracers, while reusable fluid path synthesizers that have less restrictions on fluid path material and lay-out are mainly used for research. The latest development in the field of cassette based synthesizers is the integration of reagents into the disposable cassette itself (e.g., FASTlab, GE Healthcare, Liège, Belgium) significantly reducing the work required to prepare a synthesis run. This concept also allows for quality-critical manufacturing operations like cassette and reagent assembly to be performed within a central facility. For microfluidic synthesizers all three of the concepts described above may apply: (1) using a reusable fluid path (comparable to e.g., the Advion Biosciences, Inc., Nanotek) (2) using a disposable fluid path (comparable to e.g., the Modular-Lab PharmTracer, Eckert & Ziegler Strahlen-und Medizintechnik AG, Berlin, Germany) and (3) using a disposable fluid path with reagents stored attached to or inside the disposable device (comparable to e.g. the GE FASTlab).

## 3. Functional Elements

Each functional element is designed to realize one or multiple aspects of the PET tracer synthesis process as illustrated in Figure 1. For future microfluidic PET tracer synthesizers, the engineering required to implement all required functional elements into an integral and cost effective solution has proven to be a challenge.

### 3.1. Materials and Manufacturability

Defining the fundamentals of most functional elements, the selection of microfluidic device or component materials has far-reaching consequences on design opportunities, system performance, manufacturing complexity and cost [63]. The severity of this challenge can be exemplarily illustrated by the shift from polydimethylsiloxane (PDMS)-based microfluidic PET tracer synthesizers [34,60] towards assemblies of polyether ether ketone (PEEK) with polydicyclopentadiene (pDCPD) [35,53] and a resulting fundamental redesign of functional hardware elements such as valves and reactors.

On-chip radiochemistry requires material compatibility to commonly used aggressive media such as hydrochloric acid (HCl), sodium hydroxide (NaOH), or solvents like dimethyl sulfoxide (DMSO), acetonitrile (MeCN), dimethylformamide (DMF) and ethanol (EtOH), for example. Depending on the specific PET tracer to be synthesized, required process temperatures range from –20 °C for e.g., carbon-11 chemistries [64], 20 °C for e.g., fluoride-18 radiolabeling of peptides [65], 110 °C for common PET tracers such as [^18^F]FDG [17] or [^18^F]fluoromisonidazol ([^18^F]FMISO) [66] and 160 °C for compounds such as [^18^F]FLT [18]. Recent results on microfluidic [^18^F]FLT synthesis suggest that the use of decreased reaction temperatures due to improved heat transfer in micro-scale systems is possible [67].

The levels of radioactivity processed in a practical routine range from 20 mCi (equal to 740 MBq, single patient dose) to 10 Ci (370 GBq) or more (in multi-patient dose batch production) and can lead to radiation-induced material decomposition. Resulting impurities can contaminate the product, affect the chemical process or lead to fatigue of hardware components. Due to the handling of small volumes in microfluidic systems, local activity concentrations can be significant [68]. However, an appropriate system design in terms of materials, system geometries [14] and use of a disposable fluid path may reduce the impact of radiation-induced material degradation effects.

Some glass materials may offer sufficient chemical robustness but present a cost challenge for disposable microfluidics at economy of scale [69]. Polymer materials containing fluorine-19 such as fluorinated ethylene propylene (FEP), polyvinylidene fluoride (PVDF), perfluoropolyether (PFPE) and polytetrafluoroethylene (PTFE) carry the risk of fluorine-19 contamination of fluoride-18 based radiochemistry which is widely used for radiolabeling of PET tracers worldwide today. It has been demonstrated that due to carrier addition of non-radioactive fluorine-19 from PTFE, ethylene chlorotrifluoroethylene (ECTFE, Halar^®^) and FEP, the specific activity of fluoride-18 labeled compounds is reduced [70,71,72,73]. A high specific activity is not essential for all fluorine-18 labelled tracers, for instance [^18^F]FDG, but can be an issue for tracers intended to target receptors or other saturable molecular targets.

Polydimethylsiloxane (PDMS) is widely used for the production of microfluidic devices and offers highly integrated functional components such as micro-valves [74]. However, PDMS lacks compatibility with alcohols and organic solvents as well as aqueous potassium carbonate at elevated temperatures [34,75,76]. Another disadvantage reported in literature is the diffusion of fluoride-18 into the PDMS material matrix resulting in up to 95% fluoride-18 trapping [34,77]. Additionally, it is challenging to achieve economy of scale with PDMS-based designs due to their comparably long manufacturing cycle times ranging from several minutes to hours per layer [78]. This also appears to be a road block for solvent resistant PFPE devices reported in the literature, in addition to the issue of reduced specific activity [79,80]. There are examples of microfluidic PDMS designs that have been transferred to injection moldable polymers, such as the HeatWave^TM^ TS microfluidic chip (RainDance Technologies, Inc., Billerica, MA, USA). However, such design transfers place several constraints on the functional elements of the microfluidic system and as a consequence may turn a PDMS-based prototype development into a dead end towards developing a commercial product [81].

Due to the high surface to volume ratio in microfluidic systems, positive characteristics such as improved heat transfer but also negative effects such as chemical surface impurities and the impact of residuals or dead volumes are amplified. One option to reduce material interference effects are coatings, such as poly(*p*-xylylene) polymers [82]. Whereas coatings may benefit multi-use systems, single-use architectures may show increased manufacturing complexity and cost.

With the option of material coatings excluded, Table 1 displays a short summary of polymers that could be utilized for microfluidic chip design. The illustration includes manufacturing parameters such as manufacturing method, molding cycle time and raw material cost which must be considered early in the development in order to enable a viable transfer towards commercial large scale mass production.

**Table 1:** Comparison of polymers utilized for microfluidic chip manufacturing with a view on applications in radiochemistry.

	COC Cyclic Olefin Co-Polymer	pDCPD Polydicyclopentadiene	PEEK Polyether ether ketone
Manufacturing method (single layer)	Injection molding ^1)^	Reaction injection molding ^2)^	Injection molding ^7)^
Molding cycle time (approximated)	<1 min ^1)^	>5 min ^2)^	<1 min ^7)^
Compatibility to acids	Good ^1)^	Medium ^3)^ [53]	Good ^8)^
Compatibility to bases	Good ^1)^	Good ^3)^	Good ^8)^
Compatibility to alcohols	Good ^1)^	Good ^3)^	Good ^9)^
Compatibility to acetonitrile	Good ^1)^	Good [53]	Good ^10)^
Compatibility to DMSO	Good ^1)^	Good ^4)^	Good ^10)^
Temperature Capability	150 °C ^1)^	140 °C ^5)^	134 °C ^5)^
Raw material cost (in US ¢ per gram)	~1.2 ¢/g^1)^	~1.3 ¢/g^6)^	~26.1 ¢/g ^5)^

Compiled with the friendly help of: ^1)^ TOPAS^®^ Advanced Polymers GmbH, Frankfurt-Höchst, Germany; ^2)^ Artekno Oy, Kangasala, Finland; ^3)^ Telene S.A.S., Bondues, France; ^4)^ GE Global Research, Niskayuna, NY, USA; ^5)^ MatWeb LLC, Blacksburg, VA, USA; ^6)^ Osborne Industries, Inc., Osborne, KS, USA; ^7)^ Drake Plastics LTD Co., Cypress, TX, USA; ^8)^ Tech Line Coatings, Inc., Murrieta, CA, USA; ^9)^ Zeus Inc., Orangeburg, SC, USA; ^10)^ Entegris, Inc., Billerica, MA, USA. *Financial statements are not legally binding and are subject to change.*

**Figure 2:**
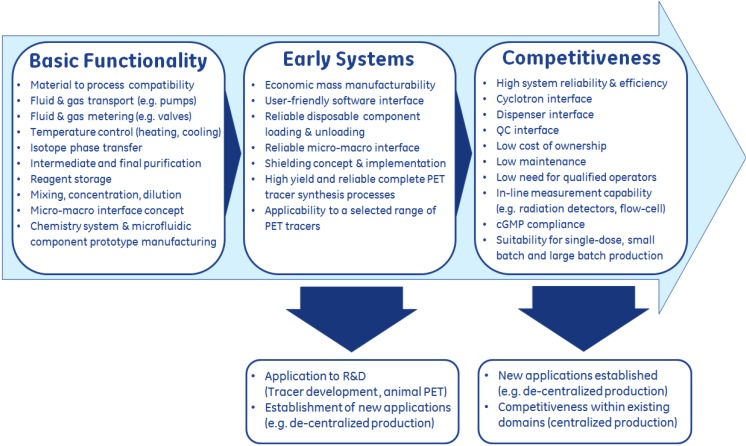
Evolution of microfluidic PET tracer synthesis systems and application to existing and new markets.

Polyether ether ketone (PEEK) has been successfully utilized for non-disposable or multiple-use systems or components in radiochemistry [53]. However, its high raw material cost is disadvantageous for single use disposable microfluidic kits. The recently reported incompatibility of polydicyclopentadiene (pDCPD) to acids as well as the long manufacturing cycle time for reaction injection molding (Figure 2) renders this particular material questionable [53]. A promising material is cyclic olefin co-polymers (COC). COC can be injection molded at economy of scale with high structure accuracy and reproducibility while maintaining cycle times below one minute [83,84]. The material is compatible to most acids, bases and solvents as well as temperatures of up to 150 °C, depending on the additives utilized (COC 6017, TOPAS^®^ Advanced Polymers GmbH, Frankfurt-Höchst, Germany). Several functional elements of today’s conventional PET tracer synthesizers such as the drying and reaction vessel of the GE FASTlab (GE Healthcare), for example, are manufactured from COC and have proven suitability for many common reagents, process conditions and regulatory requirements associated to routine PET chemistry. COC foils are available in various material grades and thicknesses that may enable the realization of on-chip membranes or contact areas for efficient heat transfer into cavities and reactors on-chip.

### 3.2. Radiation Shielding

Currently there are two basic concepts for radiation shielding of PET tracer synthesizers: The conventional “hot-cell” approach and the recently introduced “split-box” or “self-shielded” architecture [53]. Hot cells are well established installations with a weight of 4 to 8 tons providing a lead shielded work space sufficient to house one or more complete conventional PET tracer synthesizer units. The development towards more compact microfluidic synthesizers aims to accommodate an increased number of synthesis modules within one hot cell, targeting to upgrade the production capacities of existing and future PET facilities. Usually all hardware components except the control computer are located inside the hot cell, driving a high need for very compact solutions. “Split-box” or “self-shielded” architectures have recently been put into commercial practice in the Eckert & Ziegler Eurotope GmbH Modular-Lab MicroCell. In this design, the shielding is an integral part of the synthesizer system. Only hardware which is in contact with radioactive material is shielded, whereas non-contaminated elements including control electronics or pneumatic actuators are external to the shielding. This has a first advantage of significant overall system weight reduction due to elimination of the hot cell of up to 90%. The saving in weight directly translates into infrastructure cost reduction, one very important need in the field. The second advantage is the shielding of radiation sensitive hardware such as control electronics. Radiation induced hardware degradation is a common source for PET tracer synthesizer electronics failures. It limits the choice of usable electronics towards more robust and expensive components during system design and increases the need for maintenance during routine operation, especially for high activity multi-dose batch production. The challenge of “split-box” designs is the complex overall system design and, more important, the interface between shielded and non-shielded hardware elements. Every wire, cable, pneumatic or fluidic tubing and mechanical mechanism that is required at the interface must not create any radiation shine paths or leaks. On the other hand, every hardware component integrated inside the shielded compartment increases its size and weight, leading to a reduced benefit of the split-box architecture. With this challenge in mind, microfluidic systems such as EWOD chips or centrifugal microfluidics show an advantage due to their fluid transport and control technology which could eliminate the need for bulky syringe pumps and associated fluid transfer lines.

### 3.3. Fluid Transport and Control

Various liquid transport regimes have been described in literature and can be separated into: (1) capillary force driven lateral flow, (2) pressure driven platforms utilizing external or internal pressure sources such as syringe pumps, micropumps, gas expansion principles, pneumatic displacement or deformation of membranes, (3) centrifugal microfluidics actuated by rotation induced centrifugal forces, (4) electrokinetic principles utilizing electric fields gradients and forces between electric (di)poles and (5) electrowetting on dielectrics (EWOD) using electric fields to control the wetting behavior of confined droplets enabling fluid transport across a chess-board type electrode array [85,86].

Microfluidic prototypes for PET radiochemistry have been reported for the categories (2) pressure driven and (5) EWOD actuated fluid transport. For pressure driven systems, syringe pumps are often employed to transport liquids with accurate volumes and flows between functional elements in a microfluidic system. The main disadvantages of syringe pumps are their physical size, leading to an increase in shielding weight and their need for syringes connected to the microfluidic system across a connector interface. The latter is disadvantageous in the context of split-box shielding architectures and GMP requirements on sterility and cross contamination. Today, all contaminated syringes are either replaced or automatically cleaned between synthesis runs. As an alternative to syringe pumps, a pressure driven microfluidic [^18^F]FDG synthesis chip has been reported by Voccia *et al*. (Trasis SA, Ans, Belgium) utilizing on-chip membrane displacement for liquid mixing and transport [87]. This embodiment utilizes compact actuators for membrane displacement, whereas the membrane itself is integrated into the disposable fluid path.

The technically most elaborated system has been an EWOD chip for PET tracer production reported by Chen *et al*. [67]. The technology offers parallel handling of single droplets across a two dimensional plane, originally thought to be beneficial for the combination of multiple reagents for e.g., assays and multiplexing applications. Such a system could enable very compact shielding designs with the control electronics being located outside the shielding. Another benefit is the free re-programmability of on-chip processes potentially reducing the need for varying device architectures tailored to specific syntheses requirements. However, overall this technology is at an early stage of development [88].

In addition to fluid transport, appropriate fluid control may be required for steering and metering of reagents inside the microfluidic fluid path, usually requiring valve mechanisms integrated into the system. The general challenges associated to valves are reliable performance, physical size, dead volumes and the macro-to-micro interface to actuators. Conventionally, valving is facilitated by rotary motor-, stop-cock or pinch valves with limited opportunities for functional element and, sometimes more important, valve actuator miniaturization. Pneumatically or hydraulically driven, highly integrated microfluidic valves have been a big motivator for polydimethylsiloxane (PDMS)-based microfluidic systems [74]. However, after PDMS showed severe material incompatibilities to PET chemistry as explained, alternative approaches such as membrane valves utilizing chemically robust polymers gained increasing attention [89]. In contrast, EWOD and centrifugal microfluidics do not require actively driven valves and are therefore known as “valveless” architectures which may be an advantage for compact shielding architectures [90]. Nevertheless, all of the above reagent transport and control systems must go hand in hand with a corresponding reagent storage solution.

### 3.4. Reagent Storage & Release

In conventional synthesizers, PET tracer specific reagent reservoirs or kits are utilized and attached to the fluid path by means of a connector interface. The shelf-life of reagent kits is determined by the encapsulated chemicals as well as the packaging materials and architecture employed. The preferred storage vessels today are septa capped glass vials interfaced to the reactor system via needles, tubing and liquid manifold structures. Glass vials show good chemical resistance to aggressive media at low material cost while being available in various standardized sizes and shapes. However, the storage of reagents in conventional glass vials for subsequent use with microfluidic systems carries some inherent challenges: (1) Dimensions: glass vials utilized in PET chemistry contain reagent volumes in the range of 100 μL to 10 mL including a reagent excess in order to compensate for dead volumes caused by the connecting interface. In contrast, microfluidic synthesizers could process volumes below 5 μL, theoretically providing reduced consumption of potentially expensive reagents. However, this particular advantage would need to be made accessible by a corresponding reagent storage and release solution. (2) Packaging cost: apart from the pure reagent cost itself, complete kits incur high manufacturing expenses associated with filling, sealing, quality control and potential assembly of vials into carriers connected to the microfluidic reactor system. The cost per reagent vial is increasingly dominated by the cost of packaging of low reagent volumes. For the most expensive reagent in the process, the precursor, the benefit of low reagent consumption appears to be of high value in the arena of novel research compounds. However, the minimum amount of precursor packaged economically for a broad customer base may ultimately become limited by dosing accuracies and an economic minimum threshold for a yearly precursor material production at the supplier end. (3) Reliability and regulatory compliance: if the reagent dosing or vial assembly is carried out manually at point of use, it requires trained personnel and this reduces system reliability due to an increased risk of human error. Since glass vial-based reagent storage is simple, reliable and has therefore been the gold standard in radiochemistry to date, alternative approaches are rare. From a fundamental architectural point of view, expensive reagents such as the precursor could be integrated in low quantities on the microfluidic fluid path by e.g. immobilization in dedicated cavities, on channel walls or resins. The resulting reduction of transfer losses would enable “true” low reagent consumption. However, regulatory compliant packaging and associated overall system and disposable cost would need to be addressed. A reagent metering and delivery system has been reported by the van Dam group for the EWOD based PET tracer synthesizer chip, utilizing a syringe pump external to the shielding or alternatively controlled gas pressure for reagent delivery from a conventional vial to a capacitive metering element on-chip for accurate quantification and transfer of the liquids delivered across a needle interface [91]. Another approach has been reported outside the field of radiochemistry utilizing small glass ampules integrated into the microfluidic system [92]. This method enables a two-step assembly of the reagent storage solution, with the first step of filling and encapsulation of glass ampules and the second step of ampule integration into the microfluidic system. Fluids are released upon crushing of the ampules inside the microfluidic fluid path. This approach has the potential to reduce reagent transfer losses and the macro-to-micro interface complexity. However, costs associated to ampule filling, closing, microfluidic packaging and quality control would need to be commercially competitive.

A new trend in pharmaceutical packaging utilizes blister technologies and this has been reported in research as well as commercial microfluidic development projects such as the Daktari CD4 chip (Daktari Diagnostics, Inc., Cambridge, MA, USA) developed by thinXXS (thinXXS Microtechnology AG, Zweibrücken, Germany) [93]. Liquid filled blisters can be manufactured from multiple layers of metal and polymer films with tuned capabilities on chemical resistivity and moisture barriers. Challenges in the context of PET chemistry arise around reagent compatibility for strong acids and solvents across the full shelf-life time of several months as well as blister manufacturing, filling, closing and reagent release mechanisms interfaced to the microfluidic reaction system.

### 3.5. Macro-to-Micro Interface

The macro-to-micro interface is a well-known fundamental issue in microfluidics [94]. Considering chip-based microfluidic architectures for example, connections between the “lab-on-chip” and the control hardware tend to increase the risk of e.g., leaks and dead volumes causing system failures. Hence, there should be a constant design effort towards reduced macro-to-micro interface complexity. This accounts for all types of fluidic connectors, gas pressure lines, the interface to reagent storage as well as temperature control, actuators and sensors. However, the challenge is amplified by the introduction of the disposable fluid path concept requiring coupling and de-coupling of a disposable component between each synthesis run, whereas re-coupled fluid interfaces would need to meet regulatory requirements such low bioburden and low risk of cross-contamination. Comparable to existing synthesizers utilizing disposable fluid paths, integral reagent storage and preassembled fluid interfaces established within a cleanroom environment during manufacturing and packaging may become a valid route for microfluidics as well. This aspect loops back into the reagent storage question that has been discussed previously. Another approach could be the sterilization of re-coupled interfaces between each synthesis run, e.g. by means of disposable pipetting robotics. Gas lines could be sealed to the outside air by filters integrated into the disposable components, for example.

### 3.6. Mixers, Reactors and Temperature Control

PET radiochemistry utilizes short-lived radioisotopes such as ^18^F (t = 109.8 min), ^68^Ga (t = 68.3 min), ^11^C (t = 20.3 min), ^13^N (t = 9.9 min) and ^15^O (t = 2.0 min). Consequently, process times in general and chemical reaction times in particular are critical parameters for PET tracer production, depending on the specific radionuclide and PET tracer synthesis procedure of concern.

Microfluidics is dominated by laminar flow at low Reynolds numbers which results in diffusion limited mixing under the absence of specific mixing techniques. Microfluidic mixers can be categorized as “active” under the influence of an external excitation or “passive”, where contact area and contact time between two media is increased through tailored microfluidic structures [95]. In the context of PET chemistry, the most detailed study has been reported by Elizarov *et al*. on a coin-shaped microfluidic reactor for the synthesis of [^18^F]FDG [34]. Phase transfer, reagent mixing, radiolabeling and hydrolysis were carried out within a single reaction chamber. Parameters that reduced non-reacted residuals were identified as: (a) vacuum assisted turbulent-like mixing inside the reaction chamber, (b) chemically assisted mixing through a carbon dioxide yielding acid/base reaction resulting in fluid turbulence and (c) pressure assisted mixing to increase diffusion rates between highly viscous reagents.

Apart from mixing, multiple reactor structures have been reported for PET chemistry such as micro-channel or capillary-based reactors [96], highly integrated ring-shaped reactors [60] and coin-shaped reaction chambers [34]. Overall, the reactor structure should enable rapid heat transfer either through direct or capacitive heating from pre-heated reactor components [97]. Precise and rapid heating of low reaction volumes is a significant advantage of microfluidics in this field, and has demonstrated improved synthesis time and yield on some PET tracer syntheses. Heating can be carried out via resistive heaters, radiator heating and laser methods, for example, with respective design impacts on the macro-to-micro interface as well as the shielding surrounding the hardware. Microwave assisted radiochemical syntheses have been reported for over two decades yielding increased radiochemical reaction yields, better selectivity than the corresponding thermal methods and reduced reaction times [98,99,100,101,102,103,104]. However, to date this technique has found very limited application to microfluidic PET chemistry, potentially due to hardware and macro-to-micro interface integration issues as well as the lack of precise and repeatable process control. Reports on microwave transmission lines applied to polymer microfluidic reactor chips may solve the macro-to-micro interface challenge, implying constraints on the reactor geometry design for defined microwave propagation [105]. On the other hand, rapid and effective cooling may also be desired for PET tracer synthesis applications such as microfluidic carbon-11 labeling [64]. Whereas the advantage of microfluidics regarding fast heat transfer rates and low thermal capacities of small reagent volumes processed remain valid, the hardware interface must change to methods such as Peltier, gas, liquid or liquid-gas cooling, for example.

Apart from thermal energy transfer, the reactor should allow for full product recovery utilizing appropriate flow design and washing techniques [34]. Recent results by our group suggest that the reactor geometry, especially with dimensions significantly below the average positron interaction range of ~2 mm, has an impact on radiation induced radical formation and subsequent autoradiolytic product decomposition during synthesis [14].

### 3.7. Phase Transfer and Activity Concentration

Radioactive isotopes such as fluorine-18 are delivered as [^18^F]fluoride to the synthesizer in approximately 1 to 3 mL of cyclotron target water. However, commonly utilized radiolabeling approaches via nucleophilic substitution are carried out in an aprotic polar organic solvent. Hence, the radioisotopes have to undergo a phase transfer from the aqueous cyclotron target solution into a dry organic solvent. In all current generation synthesizers, this process is carried out by: (1) fluoride trapping on a silica-based resin, (2) drying of the cartridge utilizing gas and dry solvents such as acetonitrile, dimethyl sulfoxide or dimethylformamide, (3) elution of the activity utilizing an eluent containing a major fraction of an aprotic solvent (e.g., 90%), a minor fraction of water (e.g., 10%) and a phase transfer catalyst such as Kryptofix [K2.2.2] in combination with a weak base such as potassium carbonate in order to enable elution of [^18^F]fluoride into a reaction vessel [17]. The water content of the elution mixture leads often to the requirement of (4) further drying procedures such as azeotropic distillation under reduced pressure and/or elevated temperatures with gas flow or venting mechanisms for the removal of water, sometimes with repeated injections of e.g. acetonitrile. Apart from providing [^18^F]fluoride in an organic soluble form, this functional element provides activity concentration and a bridge between the macro-scale cyclotron target volume and the microfluidic processing volumes below 100 µL. A third functionality is the removal of unwanted trace level cyclotron target impurities. Several efforts have targeted to adapt this process to the micro-scale, leading to hardware challenges associated to the process control of boiling, material requirements for semi-permeable membranes or repeatability of controlled gas flows on a micro-scale. Modified and alternative phase transfer methods have been reported which can be categorized into: (1) change of chemistry, for example on the elution composition, exchange resin material and the subsequent radiolabeling method [106,107,108,109,110,111,112,113,114,115] or (2) change of hardware structures towards microfluidic dimensions [116,117,118,119], or (3) entirely new designs such as continuous flow solvent evaporation [120] or electrochemical trapping and release of radioactive species [121,122,123,124,125,126,127,128]. Recently, an increased moisture tolerance has been reported for a few microfluidic radiolabeling reactions, potentially relaxing the requirements on the drying process for several chemistries [129,130]. A combined adaptation of all methods described or a subset thereof towards new microfluidic designs may resolve the current shortcomings associated to repeatability, reproducibility, manufacturability and application to a large variety of PET tracer syntheses, turning this particular functional element into a very interdisciplinary challenge.

Whereas several of the concepts mentioned are viable within a laboratory environment, cost effective integration and mass manufacturability into potentially disposable microfluidic systems remains to be addressed. Critical topics towards mass manufacturing are cost of goods and raw material, manufacturing process complexity and its potential for automation, the resulting total chip manufacturing time (“manufacturing cycle time”) as well as low scrap rates implying a need for well controlled and reliable manufacturing methods. For example, the electrochemical method requires the integration of electrodes into the system. In early studies, only very costly materials such as glassy carbon and platinum have been used as electrode material making the approach hard to justify from a cost perspective. However, more recent work of Sadeghi *et al*. successfully employs electrode materials such as brass that may be integrated into re-usable or disposable fluid paths at reasonable manufacturing costs [128]. For resin based methods, casting processes, manual filling of cartridges and subsequent closure as well as emulsion introduction and UV curing on-chip remain questionable processes from an economy of scale perspective.

### 3.8. Intermediate and Final Purification

The majority of PET tracer syntheses use high performance liquid chromatography (HPLC) purification methods for removal of radioactive and non-radioactive impurities according to specifications stipulated by Good Manufacturing Practice (GMP) and Federal Drug Administration (FDA) regulations. New interface technologies between microfluidic PET tracer synthesis devices and HPLC equipment have been reported in literature [131], however, for routine PET tracer production there are increasing efforts to replace the footprint and complexity of HPLC equipment by simple, disposable and low cost solid phase extraction (SPE) cartridges. For [^18^F]FDG purification, the steps performed by the according SPE method are (1) removal of cationic impurities (via e.g., PS-H^+^, Macherey-Nagel GmbH & Co. KG, Düren, Germany), (2) removal of anionic impurities (via PS-HCO ^-^, Macherey-Nagel GmbH & Co. KG), (3) removal of polar impurities (via ALOX N, Macherey-Nagel GmbH & Co. KG) and (4) removal of hydrophobic impurities (via HR-P, Macherey-Nagel GmbH & Co. KG). There are growing efforts to extend SPE-based purification methods towards new tracers beyond [^18^F]FDG for a rapid transfer from a research state towards efficient production on commercial synthesizers. Successful examples are [^18^F]FLT [132], 1-α-d-(5′-deoxy-5′fluoro-(1*S*,2*R*,3*S*,4*S*)-arabinofuranosyl)-2-nitroimidazole ([^18^F]FAZA) [133], [^18^F]fluoroethyltyrosine ([^18^F]FET) [134] and [^18^F]FMISO [135]. Another approach is the application of medium pressure liquid chromatography (MPLC) utilizing medium-sized resin particles in disposable columns as a trade-off between the HPLC and SPE [136].

All of the above methods require a solid phase or resin for product trapping or retardation. Fundamentally, the small amount of reagents and reagent volumes in microfluidic systems are beneficial to the purification step since a reduced amount of solid phase material is required compared to conventional techniques. However, in addition to chemical functionality, sufficient capacity, low levels of irreversible intermediate and final product adsorption, acceptable pressure drops, tailored elution chemistries and the physical integration into microfluidic hardware imposes a challenge. Strategies for on-chip trapping of beads [60], filling of cavities [137], manual filling of tubing [61], planar alumina structures [138], UV curing of functional monoliths [119] or realization of periodic microstructures [139] have been described in literature and partially applied to PET chemistry. However, to date all of the listed resin integration methods seem to lack the compatibility to cost effective, reliable mass production, either due to specific manufacturing techniques employed (e.g., manual filling, lithography processes or liquid suspensions), limited choice of materials for hardware integration or time intensive manufacturing steps (e.g., UV curing). Hence, a purification integration technique which is compatible to microfluidic PET tracer synthesis in terms of functionality, cost effective mass production and flexibility to various purification processes required by an increasing variety of PET tracers has to be identified.

## 4. PET Tracer Production Workflow Optimization

### 4.1. Cyclotron to Quality Control Hardware Miniaturization

Taking a step back to the initial discussion on the PET tracer synthesis workflow (Figure 1), improved flow and integration between the main steps (boundary conditions) in PET tracer synthesis are important drivers of new market models such as de-centralized PET tracer production. There is a direct impact on the commercial development of microfluidic PET tracer synthesizers. In particular, these boundary conditions are the cyclotron and the quality control hardware as well as improvements on infrastructural requirements surrounding the whole workflow such as clean room and radiation shielding bunker installations. There are recent efforts towards self-shielded, miniaturized cyclotron architectures, omitting the requirement for bunkers such as the Biomarker Generator (ABT Molecular Imaging, Inc., Louisville, TN, USA) and the PETtrace 600 (prototype, GE Healthcare, Uppsala, Sweden) with an anticipated cyclotron footprint reduction of up to 50%. In addition, several commercial activities focus on the integration of PET tracer quality control (QC) from a dedicated QC laboratory setup down to a single table top device, significantly reducing the QC laboratory infrastructure burden and cost [140,141,142].

A future microfluidic synthesizer could connect the miniature cyclotron efforts with a reduced quality control setup, creating a compact, integral PET tracer production workflow that could reduce PET site cost of ownership dramatically and present a significant step towards cost effective de-centralized PET tracer production. Whereas the major commercial players in this field are capable of uniting all these efforts under one umbrella from a product portfolio perspective, the associated overall development costs are substantial and a detailed assessment on PET market needs, size and growth including reimbursement becomes a big part of the overall picture.

### 4.2. Evolution of Microfluidic Synthesizers for PET Tracer Production

Microfluidic synthesizers for PET radiopharmaceuticals are currently predominantly applied to research and development applications and will remain in this field until an acceptable level of reliability, repeatability, usability, GMP compliance and cost of ownership has been achieved (Figure 2). Whereas capillary-based systems are already approaching good utilization for PET tracer research as early systems (Figure 2), supported by a growing number of scientific publications associated to this particular architecture, hybrid or lab-on-chip microfluidic devices are currently in the stage of basic functionality development (Figure 2), due to the functional element integration challenges discussed in this review. The advantages of lab-on-chip based architectures versus capillary-based microfluidic systems are thought to impact commercial competitiveness in terms of disposable PET tracer kit production, increased usability, reduced requirements on qualified operators, GMP compliance under the benchmark of existing disposable PET tracer kit-based synthesis systems as well as potentially very compact shielding designs which relate to infrastructural costs and production flexibility.

## 5. Market Drivers for Next Generation PET Radiochemistry Platforms

A minaturized radiochemistry platform with combinations of some or all of the elements as described will achieve utility with a range of end users.

Firstly, centers involved in the research and development of novel PET tracers have demands for systems that allow radiosynthesis to take place quickly. It has been demonstrated that efficient heat transfer in microfluidic systems can give higher percentage incorporation of fluorine-18 in radiolabelling chemistries. However, as discussed in this review, complete integration with other functional elements is required for this to translate to higher isolated radiochemical yield. Furthermore, the potential for reduced consumption of radiolabelling precursors is attractive. A PET tracer with diagnostic and commercial value that can only be produced efficiently on a microfluidic scale due to inherent process synthesis requirements such as fast and precise heating, reagent concentrations or reaction kinetics would present a milestone for microfluidics in radiochemistry but has not been identified yet.

Another significant advantage will be a reduced footprint and infrastructure cost for the PET tracer manufacturing equipment and facility. The smaller physical footprint may allow a greater number of synthesis modules to be installed in current hot cells offering greater flexibility. Alternatively, self-shielded or split box designs offer more space and flexible ways to add capacity. As new PET agents will achieve regulatory approval in the future, the pressure on large scale manufacturers of PET radiopharmaceuticals will grow and urge them to include an increasing variety of new PET tracers as an offering in order to maintain commercial competitiveness. This can be eased by the introduction of new technology. Furthermore, microfluidic systems have no inherent restriction on PET tracer batch size compared to current radiochemistry modules. Indeed, advantages have been demonstrated for handling high activity levels in microfluidic geometries [14]. For PET tracers that address a large patient group size, have regulatory approval and market authorization, centralized PET tracer production is likely to remain the preferred supply chain model since the de-centralized approach will struggle to compete with the cost per dose achievable with high activity multi-dose batches produced centrally.

However, if the current PET manufacturing sites reach their maximum capacity, the cost of introducing a new tracer into the supply chain will be high, since investment in new infrastructure will be needed. Additional constraints will apply to the centralized distribution model due to the more specialized tracer diagnostics addressing decreasing patient group sizes. Hence, whereas the overall PET market may grow, the addressed patient group size for a specific tracer per hospital will decrease. This is in conflict with today’s mode of operation on “one-fits-all” tracer batch production with e.g., [^18^F]FDG, which addresses a less diversified population of patients and earns a lot of the profit based on the multi-patient per run batch production capability.

Another aspect is the potential for systems that integrate PET tracer synthesis from cyclotron to QC. It is technically feasible for this level of system integration to be realized and the aim is a reduced total cost of ownership for PET tracer manufacturing sites. If this can be accomplished, the de-centralized model may become accessible to a greater number of sites, opening the way for new markets in areas where there is no centralized PET tracer distribution network which can be low dense populated areas in developed countries as well as new markets in developing countries with infrastructural restrictions.

## 6. Conclusions

The first section of this review has illustrated the evident strengths of microfluidics for PET tracer synthesis such as reduced precursor consumption, increased synthesis efficiency and reduced system footprint. These points are supported by a steadily growing number of research publications and development projects as well as first commercial systems being available in this space. Most of the remaining risks associated to microfluidic PET technology circle around engineering challenges such as system materials, manufacturability and the integration of all functional elements required for PET tracer synthesis process execution into one, compact device. Whereas future progress towards a widespread commercial application will require substantial research and development efforts as identified by the technological needs for the separate functional system elements, the scientific and technological foundation appears to be valid and the engineering challenges generally addressable.

We propose that next generation microfluidic PET radiochemistry platforms offer more effective laboratory space utilization and synthesis flexibility for an increasing number of PET tracers. This ultimately translates into cost advantages for established manufacturing centers (centralized model) and may allow the expansion of centers manufacturing their own PET radiopharmaceuticals for approved and investigational PET agents (de-centralized production). If a high level of system integration from cyclotron to quality controlled release of PET tracers can be realized and combined with the reduced infrastructure cost of future microfluidic synthesizers, new markets in in low dense populated areas and developing countries may become commercially attractive.

Specialist centers involved in the discovery and development of new PET agents will also benefit from next generation microfluidic systems for the reasons described above and because the consumption of expensive reagents can be reduced making tracer development less expensive. Ultimately, which model grows the most will depend on total cost of ownership to run a PET tracer facility from cyclotron to quality control, the number and timeline of regulatory approvals on new PET tracers and how reimbursement in PET develops in the future.

## References

[1] Phelps M.E. (2000). Positron emission tomography provides molecular imaging of biological processes. PNAS.

[2] Audrain H. (2007). Positron emission tomography (PET) and microfluidic devices: a breakthrough on the microscale?. Angew. Chem. Int. Ed. Engl..

[3] Lu S.Y., Pike V.W., Schubiger P.A., Lehmann L., Friebe M. (2006). Micro-reactors for PET Tracer Labeling. PET Chemistry: The Driving Force in Molecular Imaging.

[4] Fortt R., Gee A. (2013). Microfluidics: a golden opportunity for positron emission tomography?. Future Med. Chem..

[5] Miller P.W. (2009). Radiolabelling with short-lived PET (positron emission tomography) isotopes using microfluidic reactors. J. Chem. Technol. Biotechnol..

[6] Miller P.W., deMello A.J., Gee A.D. (2010). Application of microfluidics to the ultra-rapid preparation of fluorine-18 labelled compounds. Curr. Radiopharm..

[7] Lucignani G. (2006). Pivotal role of nanotechnologies and biotechnologies for molecular imaging and therapy. Eur. J. Nucl. Med. Mol. Imaging.

[8] Shen C.K.-F. (2011). Microfluidic-assisted radiochemistry and PET probe synthesis. MI Gateway.

[9] Briard E., Zoghbi S.S., Siméon F.G., Imaizumi M., Gourley J.P., Shetty H.U., Lu S., Fujita M., Innis R.B., Pike V.W. (2009). Single-step High-yield Radiosynthesis and Evaluation of a Sensitive ^18^F-Labeled Ligand for Imaging Brain Peripheral Benzodiazepine Receptors with PET. J. Med. Chem..

[10] Wester H.J., Schoultz B.W., Hultsch C., Henriksen G. (2009). Fast and repetitive in-capillary production of [^18^F]FDG. Eur. J. Nucl. Med. Mol. Imaging.

[11] Pascali G., Mazzone G., Saccomanni G., Manera C., Salvadori P.A. (2010). Microfluidic approach for fast labeling optimization and dose-on-demand implementation. Nucl. Med. Biol..

[12] Liu K., Lepin E.J., Wang M.W., Guo F., Lin W.Y., Chen Y.C., Sirk S.J., Olma S., Phelps M.E., Zhao X.Z. (2010). Microfluidic-Based ^18^F-Labeling of Biomolecules for Immuno-Positron Emission Tomography. Mol. Imaging.

[13] Yokell D.L., Leece A.K., Lebedev A.A., Miraghaie R.R., Ball C.E., Zhang J.J., Kolb H.C., Elizarov A.A., Mahmood U.U. (2012). Microfluidic single vessel production of hypoxia tracer 1H-1-(3-[(18)F]-fluoro-2-hydroxy-propyl)-2-nitro-imidazole ([(18)F]-FMISO). Appl. Radiat. Isot..

[14] Rensch C., Waengler B., Yaroshenko A., Samper V., Baller M., Heumesser N., Ulin J., Riese S., Reischl G. (2012). Microfluidic reactor geometries for radiolysis reduction in radiopharmaceuticals. Appl. Radiat. Isot..

[15] Yu S. (2006). Review of ^18^F-FDG Synthesis and Quality Control. Biomed. Imaging Interv. J..

[16] Zimmermann R.G. (2013). Why are investors not interested in my radiotracer? The industrial and regulatory constraints in the development of radiopharmaceuticals. Nucl. Med. Biol..

[17] Hamacher K., Coenen H.H., Stöcklin G. (1986). Efficient stereospecific synthesis of no-carrier-added 2-[^18^F]-fluoro-2-deoxy-d-glucose using aminopolyether supported nucleophilic substitution. J. Nucl. Med..

[18] Machulla H.-J., Blocher A., Kuntzsch M., Piert M., Wei R., Grierson J. R. (2000). Simplified Labeling Approach for Synthesizing 3'-Deoxy-3'-[^18^F]Fluorothymidine ([^18^F]FLT). J. Radioanal. Nucl. Chem..

[19] Elizarov A.M. (2009). Microreactors for radiopharmaceutical synthesis. Lab Chip.

[20] Wang M.W., Lin W.Y., Liu K., Masterman-Smith M., Shen C.K.-F. (2010). Microfluidics for Positron Emission Tomography Probe Development. Mol. Imaging.

[21] Keng P.Y., Esterby M., van Dam R.M. (2012). Emerging Technologies for Decentralized Production of PET Tracers. Positron Emission Tomography - Current Clinical and Research Aspects.

[22] Liow E., O’Brien A., Luthra S., Brady F., Steel C. (2005). Preliminary studies of conducting high level production radiosyntheses using microfluidic devices. Label. Compd. Radiopharm..

[23] Steel C.J., O'Brien A.T., Luthra S. K., Brady F. (2007). Automated PET radiosyntheses using microfluidic devices. J. Label. Compd. Radiopharm..

[24] Padgett H. C., Buchanan C. R., Collier T. L., Matteo J. C., Alvord C. W. (2005). Microfluidic apparatus and method for synthesis of molecular imaging probes. U.S. Patent.

[25] Selivanova S.V., Mu L., Ungersboeck J., Stellfeld T., Ametamey S.M., Schibli R., Wadsak W. (2012). Single-step radiofluorination of peptides using continuous flow microreactor. Org. Biomol. Chem..

[26] Lu S.-Y., Watts P., Chin F.T., Hong J., Musachio J.L., Briard E., Pike V.W. (2004). Syntheses of ^11^C- and ^18^F-labeled carboxylic esters within a hydrodynamically-driven micro-reactor. Lab Chip.

[27] Miller P.W., Audrain H., Bender D., deMello A.J., Gee A.D., Long N.J., Vilar R. (2011). Rapid Carbon-11 Radiolabelling for PET Using Microfluidics. Chem. Eur. J..

[28] Gillies J.M., Prenant C., Chimon G.N., Smethurst G.J., Dekker B.A., Zweit J. (2006). Microfluidic technology for PET radiochemistry. Appl. Radiat. Isot..

[29] Wheeler T.D., Zeng D.X., Desai A.V., Onal B., Reichert D.E., Kenis P.J.A. (2010). Microfluidic labeling of biomolecules with radiometals for use in nuclear medicine. Lab Chip.

[30] Gaja V., Gomez-Vallejo V., Cuadrado-Tejedor M., Borrell JI., Llop J. (2012). Synthesis of ^13^N-labelled radiotracers by using microfluidic technology. J. Labelled Comp. Radiopharm..

[31] Simms R.W., Causey P.W., Weaver D.M., Sundararajan C., Stephenson K.A., Valliant J.F. (2012). Preparation of technetium-99m bifunctional chelate complexes using a microfluidic reactor: A comparative study with conventional and microwave labeling methods. J. Labelled Comp. Radiopharm..

[32] Haroun S., Sanei Z., Jivan S., Schaffer P., Ruth T.J., Lia P.C.H. (2013). Continuous-flow synthesis of [^11^C]raclopride, a positron emission tomography radiotracer, on a microfluidic chip. Can. J. Chem..

[33] Pascali G., Watts P., Salvadori P.A. (2013). Microfluidics in radiopharmaceutical chemistry. Nucl. Med. Biol..

[34] Elizarov A.M., van Dam M.R., Young S.S., Kolb H.C., Padgett H.C., Stout D., Shu J., Huang J., Daridon A., Heath J.R. (2010). Design and Optimization of Coin-Shaped Microreactor Chips for PET Radiopharmaceutical Synthesis. J. Nucl. Med..

[35] Bejot R., Elizarov A.M., Ball E. (2011). Batchmode microfluidic radiosynthesis of *N*-succinimidyl-4-F-18 fluorobenzoate for protein labelling. J. Labelled Comp. Radiopharm..

[36] Collier T., Akula M., Kabalka G. (2010). Microfluidic synthesis of [^18^F]FMISO. J. Nucl. Med..

[37] Lu S.-Y., Giamis A.M., Pike V.W. (2009). Synthesis of [^18^F]fallypride in a micro-reactor: rapid optimization and multiple-production in small doses for micro-PET studies. Curr. Radiopharm..

[38] Lu S.-Y., Pike V.W. (2010). Synthesis of [^18^F]xenon difluoride as a radiolabeling reagent from [^18^F]fluoride ion in a micro-reactor and at production scale. J. Fluorine Chem..

[39] Ungersboeck J., Richter S., Collier L., Mitterhauser M., Karanikas G., Lanzenberger R., Dudczak R., Wadsak W. (2012). Radiolabeling of F-18 altanserin—A microfluidic approach. Nucl. Med. Biol..

[40] Ungersboeck J., Philippe C., Mien L.K., Haeusler D., Shanab K., Lanzenberger R., Spreitzer H., Keppler B.K., Dudczak R., Kletter K., Mitterhauser M., Wadsak W. (2011). Microfluidic preparation of [^18^F]FE@SUPPY and [^18^F]FE@SUPPY:2—Comparison with conventional radiosyntheses. Nucl. Med. Biol..

[41] Ungersboeck J., Philippe C., Haeusler D., Mitterhauser M., Lanzenberger R., Dudczak R., Wadsak W. (2012). Optimization of [11C]DASB-synthesis: vessel-based and flow-through microreactor methods. Appl. Radiat. Isot..

[42] Chun J.H., Lu S., Lee Y.S., Pike V.W. (2010). Fast and High-yield Micro-reactor Syntheses of Ortho-substituted [^18^F]Fluoroarenes from Reactions of [^18^F]Fluoride Ion with Diaryliodonium Salts. J. Org. Chem..

[43] Chun J.H., Pike V.W. (2012). Selective syntheses of no-carrier-added 2- and 3-[^18^F]fluorohalopyridines through the radiofluorination of halopyridinyl(4[prime or minute]-methoxyphenyl)iodonium tosylates. Chem. Commun..

[44] Bouvet V., Wuest M., Tam P.H., Wang M., Wuest F. (2012). Microfluidic technology: An economical and versatile approach for the synthesis of *O*-(2-F-18 fluoroethyl)-ltyrosine (F-18 FET). Bioorg. Med. Chem. Lett..

[45] Bouvet V.R., Wuest M., Wiebe L.I., Wuest F. (2011). Synthesis of hypoxia imaging agent 1-(5-eoxy-5-fluoro-α-d-arabinofuranosyl)-2-nitroimidazole using microfluidic technology. Nucl. Med. Biol..

[46] Telu S., Chun J.H., Simeon F.G., Lu S., Pike V.W. (2011). Syntheses of mGluR5 PET radioligands through the radiofluorination of diaryliodonium tosylates. Org. Biomol. Chem..

[47] Pascali G., Nannavecchia G., Pitzianti S., Salvadori P.A. (2011). Dose-on-demand of diverse ^18^F-fluorocholine derivatives through a two-step microfluidic approach. Nucl. Med. Biol..

[48] Anderson H., Pillarsetty N., Cantorias M., Lewis J.S. (2010). Improved synthesis of 2′-deoxy-2′-[^18^F]-fluoro-1-β-d-arabinofuranosyl-5-iodouracil ([^18^F]-FIAU). Nucl. Med. Biol..

[49] Philippe C., Ungersboeck J., Schirmer E., Zdravkovic M., Nics L., Zeilinger M., Shanab K., Lanzenberger R., Karanikas G., Spreitzer H. (2012). [^18^F]FE@SNAP—A new PET tracer for the melanin concentrating hormone receptor 1 (MCHR1): microfluidic and vessel-based approaches. Bioorg. Med. Chem..

[50] Dahl K., Schou M., Halldin C. (2012). Radiofluorination and reductive amination using a microfluidic device. J. Labelled Compd. Radiopharm..

[51] Kealey S., Plisson C., Collier T.L., Long N.J., Husbands S.M., Martarello L., Gee A.D. (2011). Microfluidic reactions using [11C]carbon monoxide solutions for the synthesis of a positron emission tomography radiotracer. Org. Biomol. Chem..

[52] Richter S., Bouvet V., Wuest M., Bergmann R., Steinbach J., Pietzsch J., Neundorf I., Wuest F. (2012). ^18^F-Labeled phosphopeptide-cell-penetrating peptide dimers with enhanced cell uptake properties in human cancer cells. Nucl. Med. Biol..

[53] Lebedev A., Miraghaie R., Kotta K., Ball C.E., Zhang J., Buchsbaum M.S., Kolb H.C., Elizarov A. (2013). Batch-reactor microfluidic device: first human use of a microfluidically produced PET radiotracer. Lab Chip.

[54] Gillies J.M., Prenant C., Chimon G.N., Smethurst G.J., Perrie W., Hamblett I., Dekker B., Zweit J. (2006). Microfluidic reactor for the radiosynthesis of PET radiotracers. Appl. Radiat. Isot..

[55] Ball C.E., Diener L.T., Elizarov A.M., Ford S., Kolb H.C., Miraghaie R., van Dam R.M., Zhang J. (2011). Microfluidic radiosynthesis system for positron emission tomography biomarkers. U.S. Patent.

[56] Zeng D., Desai A.V., Ranganathan D., Wheeler T.D., Kenis P.J.A., Reichert D.E. (2013). Microfluidic radiolabeling of biomolecules with PET radiometals. Nucl. Med. Biol..

[57] Miller P.W., Long N.J., de Mello A.J., Vilar R., Passchier J., Gee A. (2006). Rapid formation of amides via carbonylative coupling reactions using a microfluidic device. Chem. Commun. (Camb.).

[58] Miller P.W., Jennings L.E., deMello A.J., Gee A.D., Long N.J., Vilar R. (2009). A microfluidic approach to the rapid screening of palladium-catalysed aminocarbonylation reactions. Adv. Synth. Catal..

[59] Arima V., Pascali G., Lade O., Kretschmer H., Bernsdorf I., Hammond V., Watts P., de Leonardis F., Tarn M., Pamme N. (2013). Radiochemistry on chip: towards dose-on-demand synthesis of PET radiopharmaceuticals. Lab Chip.

[60] Lee C., Sui G., Elizarov A.M., Shu C.J., Shin Y., Dooley A.N., Huang J., Daridon A., Wyatt P., Stout D. (2005). Multistep Synthesis of a Radiolabeled Imaging Probe Using Integrated Microfluidics. Science.

[61] Keng P.Y., Chen S., Ding H., Sadeghi S., Shah G.J., Dooraghi A., Phelps M.E., Satyamurthy N., Chatziioannou A.F. (2012). Micro-chemical synthesis of molecular probes on an electronic microfluidic device. PNAS.

[62] Kim H.K., Chen S., Javed M.R., Lei J., Kim C.-J., Keng P.Y., van Dam R.M. Multi-step organic synthesis of four different molecular probes in digital microfluidic devices. Proceedings of International Conference on Miniaturized Systems for Chemistry and Life Sciences (mTAS).

[63] Le S.J., Sundararajan N. (2010). Microfabrication for Microfluidics.

[64] Kinzl M., Lade O., Schultz C.P., Steckenborn A., Thalmann F. (2010). Method for producing a radiopharmaceutical.

[65] Wängler C., Niedermoser S., Chin J., Orchowski K., Schirrmacher E., Jurkschat K., Iovkova-Berends L., Kostikov A.P., Schirrmacher R., Wängler B. (2012). One-step (18)F-labeling of peptides for positron emission tomography imaging using the SiFA methodology. Nat. Protoc..

[66] Patt M., Kuntzsch M., Machulla H.-J. (1999). Preparation of [^18^F]fluoromisonidazole by nucleophilic substitution on THP-protected precursor: Yield dependence on reaction parameters. J. Radioanal. Nucl. Chem..

[67] Chen S., Javed R., Lei J., Kim H.-K., Flores G., van Dam R. M., Keng P. Y., Kim C.-J. Synthesis of diverse tracers on EWOD microdevice for positron emission tomography (PET). Technical Digest of the Solid-State Sensor and Actuator workshop.

[68] Zacheo A., Arima V., Pascali G., Salvadori P., Zizzari A., Perrone E., de Marco L., Gigli G., Rinaldi R. (2011). Radioactivity resistance evaluation of polymeric materials for application in radiopharmaceutical production at microscale. Microfluid. Nanofluid..

[69] Fiorini G.S., Daniel T. (2005). Disposable microfluidic devices: fabrication, function, and application. BioTechniques.

[70] Füchtner F., Preusche S., Mäding P., Zessin J., Steinbach J. (2008). Factors affecting the specific activity of [^18^F]fluoride from a [^18^O]water target. Nuklearmedizin.

[71] Link J.M., Shoner S.C., Krohn K.A. (2012). Sources of carrier F-19 in F-18 fluoride. AIP Conf. Proc..

[72] Lapi S.E., Welch M.J. (2012). A historical perspective on the specific activity of radiopharmaceuticals: What have we learned in the 35 years of the ISRC?. Nucl. Med. Biol..

[73] Berridge M. S., Apana S. M., Hersh J. M. (2009). Teflon radiolysis as the major source of carrier in fluorine-18. J. Label Compd. Radiopharm..

[74] Unger M.A., Chou H.P., Thorsen T., Scherer A., Quake S.R. (2000). Monolithic microfabricated valves and pumps by multilayer soft lithography. Science.

[75] Sollier E., Murray C., Maoddi P., Di Carlo D. (2011). Rapid prototyping polymers for microfluidic devices and high pressure injections. Lab Chip.

[76] Lee J.N., Park C., Whitesides G.M. (2003). Solvent Compatibility of Poly(dimethylsiloxane)-Based Microfluidic Devices. Anal. Chem..

[77] Elizarov A.M., Ball C.E., Zhang J., Kolb H.C., Van Dam M.R., Diener L., Ford S., Miraghaie R. (2011). Portable Microfluidic Radiosynthesis System for Positron Emission Tomography Biomarkers and Program Code. U.S. Patent Application.

[78] Mukhopadhyay R. (2007). When PDMS isn’t the best. Anal. Chem..

[79] Huang Y., Castrataro P., Lee C.C., Quake S.R. (2007). Solvent resistant microfluidic DNA synthesizer. Lab Chip.

[80] Rolland J.P., van Dam R.M., Schorzman D.A., Quake S.R., De Simone J.M. (2004). Solvent resistant photocurable “Liquid Teflon” for micro fluidic device fabrication. J. Am. Chem. Soc..

[81] Koranda M. (2012). Kunststoff-Know how: Basis für Lab-on-Chips zur Zielgen-Anreicherung. Laborwelt.

[82] Sasakia H., Onoe H., Osakia T., Kawanoa R., Takeuchia S. (2010). Parylene-coating in PDMS microfluidic channels prevents the absorption of fluorescent dyes. Sens. Act. B Chem..

[83] Steigert J., Haeberle S., Brenner T., Mueller C., Steinert C.P., Koltay P., Gottschlich N., Reinecke H., Rühe J., Zengerle R., Ducree J. (2007). Rapid prototyping of microfluidic chips in COC. J. Micromech. Microeng..

[84] Attia U. M., Marsona S., Alcockb J. R. (2009). Micro-Injection moulding of polymer microfluidic devices. Microfluid. Nanofluid..

[85] Mark D., Haeberle S., Roth G., von Stetten F., Zengerle R., Kakaç S., Kosoy B., Li D., Pramuanjaroenkij A. (2010). Microfluidic Lab-on-a-Chip Platforms: Requirements, Characteristics and Applications. Microfluidics based microsystems—Fundamentals and Applications.

[86] Haeberle S., Zengerle R. (2007). Microfluidic platforms for lab-on-a-chip applications. Lab Chip.

[87] Voccia S., Morelle J., Aerts J., Lemaire C., Luxen A., Phillipart G. (2009). Mini-fluidic chip for the total synthesis of PET tracers. J. Labelled Comp. Radiopharm..

[88] Fair R.B. (2007). Digital microfluidics: is a true lab-on-a-chip possible?. Microfluid. Nanofluid..

[89] Rensch C., Wängler B., Boeld C., Baller M., Samper V., Heumesser N., Ehrlichmann W., Riese S., Reischl G. (2011). [^18^F]FMISO Synthesis on a chip-based microfluidic research platform. J. Nucl. Med..

[90] Gorkin R., Park J., Siegrist J., Amasia M., Lee B.S., Park J.M., Kim J., Kim H., Madou M., Cho Y.K. (2010). Centrifugal microfluidics for biomedical applications. Lab Chip.

[91] Ding H., Sadeghi S., Shah G.J., Chen S., Keng P.Y., Kim C.J., van Dam R.M. (2012). Accurate dispensing of volatile reagents on demand for chemical reactions in EWOD chips. Lab Chip.

[92] Hoffmann J., Mark D., Lutz S., Zengerle R., von Stetten F. (2010). Pre-storage of liquid reagents in glass ampoules for DNA extraction on a fully integrated lab-on-a-chip cartridge. Lab Chip.

[93] Disch A., Mueller C., Reinecke H. (2007). Low Cost Production of Disposable Microfluidics by Blister Packaging Technology. Conf. Proc. IEEE Eng. Med. Biol. Soc..

[94] Fredrickson C.K., Fan Z.H. (2004). Macro-to-micro interfaces for microfluidic devices. Lab Chip.

[95] Lee C.Y., Chang C.L., Wang Y.N., Fu L.M. (2011). Microfluidic Mixing: A Review. Int. J. Mol. Sci..

[96] Brady F., Luthra S.K., Gillies J.M., Geffery N.T. (2003). Use of microfabricated devices. U.S. Patent.

[97] Samper V., Riese S., Rensch C., Boeld C., Reischl G., Heumesser N., Baller M. (2009). Numerical simulation of heat transfer in conventional *vs.* microfluidic reactors and experimental benefits for [^18^F]PET tracer synthesis. 9th World Molecular Imaging Congress (WMIC).

[98] Stone-Elander S., Elander N. (2002). Microwave application in radiolabeling with short-lived positron-emitting radionuclides. J. Label. Compd. Radiopharm..

[99] Guo N., Alagille D., Tamagnan G., Price R.R., Baldwin R.M. (2008). Microwave-induced nucleophilic [^18^F]fluorination on aromatic rings: Synthesis and effect of halogen on [^18^F]fluoride substitution of meta-halo (F, Cl, Br, I)-benzonitrile derivatives. Appl. Rad. Isot..

[100] Mandap K. S., Ido T., Kiyono Y., Kobayashi M., Lohith T. G., Mori T., Kasamatsu S., Kudo T., Okazawa H., Fujibayashi Y. (2009). Development of microwave-based automated nucleophilic [^18^F]fluorination system and its application to the production of [^18^F]flumazenil. Nucl. Med. Biol..

[101] Scott P.J.H., Shao X. (2010). Fully automated, high yielding production of *N*-succinimidyl 4-[^18^F]fluorobenzoate ([^18^F]SFB), and its use in microwave-enhanced radiochemical coupling reactions. J. Label. Compd. Radiopharm..

[102] Hou S., Phung D.L., Lin W.Y., Wang M.W., Liu K., Shen C.K. (2011). Microwave-assisted one-pot synthesis of N-succinimidyl-4[^18^F]fluorobenzoate ([^18^F]SFB). J. Vis. Exp..

[103] Kumar P., Wiebe L.I., Asikoglu M., Tandon M., McEwan A.J. (2002). Microwave-assisted (radio)halogenation of nitroimidazole-based hypoxia markers. Appl. Radiat. Isot..

[104] Hwang D.R., Moerlein S.M., Welch M.J. (1989). Microwave-facilitated synthesis of [^18^F]-Spiperone. J. Lab. Compd. Radiopharm..

[105] Oh K., Sklavounos A.H., Marchiarullo D.J., Barker N.S., Landers J.P. Microwave-assisted polymerade chain reaction (PCR) in disposable microdevices. 15th International Conference on Miniaturized Systems for Chemistry and Life Sciences.

[106] Seo J.W., Lee B.S., Lee S.J., Oh S.J., Chi D.Y. (2011). Fast and Easy Drying Method for the Preparation of Activated [^18^F]Fluoride Using Polymer Cartridge. Bull. Korean Chem. Soc..

[107] Lee S.J., Oh S.J., Chi D.Y., Kang S.H., Kil H.S., Kim J.S., Moon D.H. (2007). One-step high-radiochemical-yield synthesis of [^18^F]FP-CIT using a protic solvent system. Nucl. Med. Biol..

[108] Kim D.W., Ahn D.S., Oh Y.H., Lee S., Kil H.S., Oh S.J., Lee S.J., Kim J.S., Ryu J.S., Moon D.H. (2006). A New Class of SN2 Reactions Catalyzed by Protic Solvents: Facile Fluorination for Isotopic Labeling of Diagnostic Molecules. Am. Chem. Soc..

[109] Wester H.J., Henriksen G., Wessmann S. (2011). Method for the direct elution of reactive [^18^F]fluoride from an anion exchange resin in an organic medium suitable for radiolabelling without any evaporation step by the use of alkalimetal and alkaline earth metal cryptates.

[110] Wessmann S.H., Henriksen G., Wester H.J. (2012). Cryptate mediated nucleophilic ^18^F-fluorination without azeotropic drying. Nuklearmedizin.

[111] Eshima D.B., Husnu M., Padgett H., Klausing T.A., Bouton C.E., Benecke H., Garbark D.B. (2012). Methods and compositions for drying in the preparation of radiopharmaceuticals.

[112] Lemaire C., Voccia S., Aerts J., Luxen A., Morelle J.L., Philipart G. (2008). Method for the direct elution of reactive ^18^f fluoride from an anion exchange resin in an organic medium suitable for radiolabelling without any evaporation step by the use of strong organic bases.

[113] Toorongian S.A., Mulholland G.K, Jewett D.M., Bachelor M.A., Kilbourn M.R. (1990). Routine production of 2-deoxy-2-^18^F fluoro-dglucose by direct nucleophilic exchange on a quaternary 4-aminopyridinium resin. Int. J. Rad. Appl. Instrum. B.

[114] Aerts J., Voccia S., Lemaire C., Giacomelli F., Goblet D., Thonon D., Plenevaux A., Warnock G., Luxen A. (2010). Fast production of highly concentrated reactive [^18^F] fluoride for aliphatic and aromatic nucleophilic radiolabelling. Tetrahedron. Lett..

[115] Voccia S., Aerts J., Lemaire C., Luxen A., Morelle J. L., Philippart G. (2007). Method for the preparation of reactive [^18^F]fluoride.

[116] Fortt R., Liow E., Riese S., Steel C. (2007). Nucleophilic radiofluorination using microfabricated devices.

[117] De Leonardis F., Pascali G., Salvadori P.A., Watts P., Pamme N. (2011). On-chip pre-concentration and complexation of [^18^F]fluoride ions via regenerable anion exchange particles for radiochemical synthesis of Positron Emission Tomography tracers. J. Chromatogr. A.

[118] De Leonardis F., Pascali G., Salvadori P.A., Watts P., Pamme N. (2010). Microfluidic modules for [^18^F-] activation - Towards an integrated modular lab on a chip for PET radiotracer synthesis. Proc. MicroTAS.

[119] Ismail R., Park K.-J., van Dam M.R., Keng P. (2012). Functional polymer monoliths towards solid phase radiosynthesis on microfluidic chip. J. Nucl. Med..

[120] Cvetkovic B., Lade O., Marra L., Arima V., Rinaldi R., Dittrich P.S. (2012). Nitrogen supported solvent evaporation using continuous flow microfluidics. RSC Adv..

[121] Hamacher K., Hirschfelder T., Coenen H.H. (2002). Electrochemical cell for separation of [^18^F]fluoride from irradiated ^18^O-water and subsequent no carrier added nucleophilic fluorination. Appl. Radiat. Isot..

[122] Hamacher K., Coenen H.H. (2006). No-carrier-added nucleophilic ^18^F-labelling in an electrochemical cell exemplified by the routine production of [^18^F]altanserin. Appl. Radiat. Isot..

[123] Alexoff D., Schlyer D.J., Wolf A.P. (1989). Recovery of [^18^F]fluoride from [^18^O]water in an electrochemical cell. Int. J. Rad. Appl. Instrum. A.

[124] Baller M., Samper V., Rensch C., Boeld C. (2010). Elechtrochemical Phase Transfer Devices and Methods.

[125] Wong R., Iwata R., Saiki H., Furumoto S., Ishikawa Y., Ozeki E. (2012). Reactivity of electrochemically concentrated anhydrous F-18 fluoride for microfluidic radiosynthesis of F-18-labeled compounds. Appl. Radiat. Isot..

[126] Saiki H., Iwata R., Nakanishi H., Wong R., Ishikawa Y., Furumoto S., Yamahara R., Sakamoto K., Ozeki E. (2010). Electrochemical concentration of no-carrier-added [(18)F]fluoride from [(18)O]water in a disposable microfluidic cell for radiosynthesis of (18)F-labeled radiopharmaceuticals. Appl. Radiat. Isot..

[127] Saito F., Nagashima Y., Goto A., Iwaki M., Takahashi N., Oka T., Inoue T., Hyodo T. (2007). Electrochemical transfer of ^18^F from ^18^O water to aprotic polar solvent. Appl. Radiat. Isot..

[128] Sadeghi S., Liang V., Cheung S., Woo S., Wu C., Ly J., Deng Y., Eddings M., van Dam R.M. (2013). Reusable electrochemical cell for rapid separation of [^18^F]fluoride from [^18^O]water for flow-through synthesis of ^18^F-labeled tracers. Appl. Radiat. Isot..

[129] Chun J.H., Pike V.W. (2012). Single-step radiosynthesis of “^18^F-labeled click synthons” from azide-functionalized diaryliodonium salts. Eur. J. Org. Chem..

[130] Pascali G., Pitzianti S., Del Carlo S., Saccomanni G., Manera M., Macchia M. (2011). Initial studies on the effect of water in microfluidic radiofluorinations. J. Labelled Compnd. Radiopharm..

[131] Shah G.J., Lei J., Chen S., Kim C.-J., Keng P.Y., van Dam R.M. Automated injection from EWOD digital microfluidic chip into HPLC purification system. Proceedings of International Conference on Miniaturized Systems for Chemistry and Life Sciences (mTAS).

[132] Nandy S.K., Rajan M.G.R. (2010). Fully automated and simplified radiosynthesis of [^18^F]-3′-deoxy-3′-fluorothymidine using anhydro precursor and single neutral alumina column purification. J. Radioanal. Nucl. Chem..

[133] Nandy S.K., Rajan M.G.R. (2010). Simple, column purification technique for the fully automated radiosynthesis of [^18^F]fluoroazomycinarabinoside ([^18^F]FAZA). Appl. Radiat. Isot..

[134] Zuhayra M., Alfteimi A., Forstner C.V., Lützen U., Meller B., Henze E. (2009). New approach for the synthesis of [^18^F]fluoroethyltyrosine for cancer imaging: simple, fast, and high yielding automated synthesis. Bioorg. Med. Chem..

[135] Wang M., Zhang Y., Zhang Y., Yuan H. (2009). Automated synthesis of hypoxia imaging agent [^18^F]FMISO based upon a modified Explora FDG4 module. J. Radioanal. Nucl. Chem..

[136] Baller M., De Marco E., Dumont P., Fortt R., Franci X., Kuci S., Samper V., Steel C. (2010). Chromatography components.

[137] Tarn M.D., Pascali G., De Leonardis F., Watts P., Salvadori P.A., Pamme N. (2013). Purification of 2-[^18^F]fluoro-2-deoxy-d-glucose by on-chip solid-phase extraction. J. Chromatogr. A..

[138] Chen S., Lei J., van Dam R.M., Keng P.Y., Kim C.-J. Planar alumina purification of ^18^F-labeled radiotracer synthesis on EWOD chip for positron emission tomography (PET). 16th International Conference on Miniaturized Systems for Chemistry and Life Sciences (mTAS).

[139] Billen J., Desmet G. (2007). Understanding and design of existing and future chromatographic support formats. J. Chromatogr. A..

[140] Eshima D., Husnu M., Stone J. (2012). Method and system for automated quality control platform.

[141] Janus A., Ketzscher U. Automatic quality control for PET and MI tracers. http://www.qc1.com/.

[142] Chen S., Ly J., van Dam R.M. (2013). Towards miniaturized quality control for ^18^F-labeled PET tracers: Separation of D-FAC alpha and beta anomers via capillary electrophoresis. J. Nucl. Med..

